# Postural Adaptations in Preadolescent Karate Athletes Due to a One Week Karate Training Camp

**DOI:** 10.2478/hukin-2013-0044

**Published:** 2013-10-08

**Authors:** Stefano Vando, Davide Filingeri, Lucio Maurino, Helmi Chaabène, Antonino Bianco, Gianluca Salernitano, Calogero Foti, Johnny Padulo

**Affiliations:** 1 Faculty of Medicine and Surgery, University of “Tor Vergata” Rome, ITALY.; 2 Environmental Ergonomics Research Center, Loughborough University, LE11 3TU, UK.; 3 Faculty of Medicine and Surgery, University of “Federico II” Napoli, ITALY.; 4 Higher Institute of Sport and Physical Education of Ksar Saïd, University of Manouba, Tunis, TUNISIA.; 5 Tunisian Research Laboratory “Sports Performance Optimization” National Center of Medicine and Science in Sport, Tunis, TUNISIA.; 6 Sport and Exercise Sciences Research Unit University of Palermo, Palermo, ITALY.; 7 Italian Society for Posture and Gait Research (ISPG) Caserta, ITALY.; 8 Physical and Rehabilitation Medicine, Tor Vergata University, Rome, ITALY.

**Keywords:** physical exercise, karate training, training and testing, centre of pressure, postural sway, proprioception

## Abstract

The aim of this study was to investigate the effect of an increasing number of training hours of specific high-intensity karate training on postural sway in preadolescent karate athletes. Seventy-four karatekas were randomly assigned to 1 of 2 groups: Karate Group (KG=37): age 10.29±1.68 yrs; or Control Group (CG= 37): age 10.06±1.77 yrs. The KG performed two sessions per day for 1 week in total, while the CG performed only 3 sessions during the same period. The center-of-pressure length (COPL) and velocity (COPV) were recorded under four different experimental conditions: open eyes (EO), closed eyes (EC), open eyes monopodalic left (EOL), open eyes monopodalic right (EOR), pre as well as post training intervention. Post-camp results indicated significant differences between the groups in the COPL p<0.001; an interaction of training type×time in the COPV (p<0.001) and an interaction of training type×time (p=0.020). The KG revealed an improvement in the COPL from pre to post-camp under conditions of EO [−37.26% (p<0.001)], EC [−31.72% (p<0.001)], EOL [−27.27% (p<0.001)], EOR [−21.44% (p<0.001)], while CG revealed small adaptations in conditions of EO (3.16%), EC (0.93%), EOL (−3.03%), EOR (−0.97%). Furthermore, in the KG an improvement in the COPV from pre to post-camp was observed in conditions of EO [−37.92% (p<0.001)], EC [−32.52% (p<0.001)], EOL [−29.11% (p<0.001)], EOR [−21.49% (p<0.001)]. In summary, one-week of high intensity karate training induced a significant improvement in static body balance in preadolescent karate athletes. Karate performance requires high-levels of both static and dynamic balance. Further research dealing with the effect of karate practice on dynamic body balance in young athletes is required.

## Introduction

The World Karate Federation estimates that there is over 10 million athletes and 100 million karate practitioners worldwide (World Karate, 2013). This makes karate one of the most popular martial art/combat sport (Koropanovski et al., 2012). However, although there is no many sport disciplines that can attract such high participation, karate has only recently driven the interest of the scientific community to the effects of its longstanding practice on human performance. Therefore, further research is still needed to clarify e.g. the energetic demand of different types of competitions (kata vs. kumite), the differences between male and female athletes as well as the impact of specialized practice (kata vs. kumite competitors) (Chaabene et al., 2012). One of the most investigated topics in studies focusing on karate performance is related to the effects of this type of activity on postural changes.

It has been reported that balance, either static (i.e., stationary body) or dynamic (i.e., while moving) is of utmost importance for optimizing and developing athlete’s fundamental motor skills (Violan et al., 1997). It has been established that sport disciplines, particularly those requiring skilled and fast actions, improve postural control (Leong et al., 2011; Babiloni et al., 2010; Del Percio et al., 2007b; Pau et al., 2012). In this context, karate has been shown to represent an effective stimulus to improve balance control (Filingeri et al., 2012). This seems to result from a regular and intense practice of complex motor tasks, producing a significant load to the ankle joint. Combat sport athletes show a better use of the biomechanical properties of the lower limbs than non-athletes (Lan et al., 2002; Perrin et al., 2002). However, in almost all the studies focusing on karate and postural control (except that of Viloan et al. (1997)), only adult subjects were tested (Filingeri et al., 2012).

Although this could be due to ethical difficulties in recruiting research groups consisting of individuals under 18 years of age, the lack of studies involving preadolescent subjects is surprising, particularly if considering that youth comprises 65% of karate practitioners worldwide (WKF, 2012). Violan et al. (1997), in a study conducted with young boys (8 to 14 years old), revealed that six months of karate training (twice a week) induced a greater improvement in balance (Falamingo test eyes closed) when compared to other pre-adolescents involved in recreational sports. The postural-sway performance represents an indicator of postural and balance control ability, which is considered a key determinant of sport performance and susceptibility to injuries. Although there is extensive literature on the superior postural ability of adult athletes when compared to non-athletes (Hrysomallis, 2011), overall, there is a lack of knowledge regarding the intermediate phase of the postural development, e.g. in preadolescent athletes (Biec and Kuczynski, 2010), particularly in those who practice martial arts such as karate.

The relationship between balance ability and athletic performance is still unclear (Hrysomallis, 2011). Increasing our understanding of this relationship, and how the impact of a specific sports discipline can influence the development of postural control, is of high interest for sports performance, as well as for rehabilitation and clinical purposes. Moreover, participating in summer training camps, consisting of a one week intensive training program, is widely practiced amongst karatekas. For these reasons, in the present study, the postural-sway performance of 74 preadolescent males who had practiced karate on a regular basis, during the 4 years before this study, was investigated. Specifically, the effects of a short term increase in the volume of training in preadolescent karatekas was investigated and compared between a group participating in a one week intensive karate training camp (14 training sessions during one week), and a group performing a regular training schedule (3 training sessions during one week). The experimental design aimed at a comparison between the two groups in terms of the pre and post-training camp postural-sway performance. To our knowledge this represents the first study which investigated the impact of such a training stimulus on balance control in preadolescent karatekas.

## Material and Methods

### Participants

This study was conducted with the voluntary participation of 74 young male karatekas who were randomly assigned to either an experimental group (KG = 37: means ± *SD*: age 10.29±1.68 years, body height 1.42±0.11 m, body mass 38.04±10.45 kg, BMI 18.98±4.21 kg·m^−2^) or a control group (CG = 37: means ± *SD*: age 10.06±1.77 years, body height 1.39±0.11 m, body mass 37.81±11.29 kg, BMI 18.62±4.02 kg·m^−2^). The inclusion criteria were as follows: 4 years of training experience in karate with regular training sessions 2–3 times a week (total 3 – 4 hours per-week) and no experience with Wii Fit videogame. The participants were healthy without any muscular, neurological, and tendineous injury. Both groups were homogeneous with regard to their training status. None of the participants had undergone any endurance strenuous activity and resistance training outside of their normal training protocol. The study design conformed to the Declaration of Helsinki 1964 and was conducted after approval from the local Ethics Committee. The procedures, risks and goals were explained to the participants and their parents, and written parental consent was obtained prior to commencement of the study.

### Procedure

Before and after the training intervention, all participants, underwent a battery of postural stability tests consisting of 4×30-s recordings (Biec and Kuczynski, 2010). Four standing balance tasks were chosen based on their characteristics and common use in previous studies (Bauer et al., 2008; Springer et al., 2007). The balance tests included: 1) open eyes (EO), 2) closed eyes (EC) (feet were kept parallel and shoulders width apart), 3) closed eyes monopodalic left (EOL), 4) open eyes monopodalic right (EOR). These were performed in a random order (Latin square design) with 1 minute of recovery between trials. During each trial the participants were instructed to keep their hands on their hips and to remain as still as possible. Data were collected for 30-s during double as well as single limb trials. All tests were performed on Nintendo™ Wii Balance Board (WBB). WBB had been previously validated by Clark et al. (2010). This device contains 4 micro foil-type strain gauge transducers that are located in each of the 4 corners of the board with sampling rate of 100 Hz (see Scoppa et al. (2013) for minimal sampling rates for postural investigations i.e. 60 Hz). The WBB was interfaced to a laptop computer using a custom-written software CoreMeter™ 0.9 (Latina, Italy) and calibrated by placing a variety of known loads at different positions on the board (Vando et al., 2013).

During testing procedures, the device could be interrogated at any time to read the current settings from the four strain-gauge sensors on the board, which were delivered as 16-bit integers (Clark et al., 2010). By taking into account the position of the sensors and the values recorded, the position of the COP could be easily calculated. The WBB sensors have an internal fixed sampling rate, which we determined to be of 100 Hz. Raw calibration data and raw sensor values were stored in a relational database on the local machine. This allowed post-test processing of data. A report generation tool was used to analyze the data collected and summary reports were produced (Hubbard et al., 2012).

In this study, the outcome measure was the total of the center of pressure (COP) path length. Given that the trials were performed on a fixed time interval, the COP path length (COPL - mm) recorded was analogous to a measure of the average COP velocity (COPV - mm·s^−1^). Therefore total COP path length was chosen as the primary outcome measure because it is known to be a reliable and valid measure of standing balance (Clark et al., 2010).

### Training intervention

The KG performed two 60 min training sessions per day (morning and afternoon) for seven days during the Summer Camp. The total training volume for the KG was of 14 h. The training routine performed during the morning and afternoon training sessions consisted of: 10 minutes of coordinative trunk, arms and legs exercises in different body planes at a distance of 15-m (in shuttle); 15 minutes of press-ups, lunges and extra-rotations, lower limb exercises in different body planes followed by specific static-dynamic stretching postures performed in standing and ground positions; 35 minutes of Karate kick exercises.

The CG performed the same training routine as the KG group. However, this differed in total volume, as it was performed only for three days (during the same week of the summer camp). The total training volume for the CG was of 3 h. During the training camp all the athletes lived together and followed a diet provided by a sports nutritionist.

### Statistical analysis

Data were tested for normality using the Shapiro-Wilk normality test. The variables studied were: COP path length (i) and COP velocity (ii) in four different trials (EO, EC, EOL, EOR). These were analysed by a two-way repeated measures ANOVA and adjusted using the *Bonferroni’s* correction. The sample size was determined with post hoc statistical power analysis with G-Power 3.1.3. Using the statistical power of ANOVA by SPSS we calculated the total sample size with G-Power 3.1.3. The effect size was calculated for all variables between pre and post-testing. The threshold for small, moderate and large effect were 0.20, 0.50 and 0.80, respectively (Cohen, 1988). For testing the repeatability of the measure, we performed an Intra-class Correlation Coefficient (ICC) within length and velocity of the COP before the start of this study as a standard quality assessment procedure. The within factor was the time with two levels (pre and post-training) and the between factor was the training design with two levels (KG and CG).

The *t*-student test for independent samples was used to detect any initial differences between groups. The level of statistical significance was set at *p* ≤ 0.05 in all comparisons. Data were analyzed using XLSTAT 12.3.01 (Addinsoft, SARL, New York) statistical software package. Descriptive statistics were expressed as mean ± *SD*.

## Results

All data are reported in Table 1. There was no difference between groups at baseline conditions for age, body height, body mass, BMI, training experience and all variables studied. Length and velocity of the COP showed highly reliable data, with an ICC as a standard quality assessment procedure on COPL (range 0.89 – 0.97) and COPV (range 0.88 – 0.97).

Data from the KG revealed a significant improvement of COPL with respect to baseline conditions in EO −37.26% (*p* < 0.001), EC −31.72% (*p* < 0.001), EOL −27.27% (*p* < 0.001), EOR −21.44% (*p* < 0.001). Data from the CG revealed a small change in EO 3.16%, EC 0.93%, EOL −3.03%, EOR −0.97%. COPV presented a high significant improvement in the KG with respect to baseline conditions in EO −37.92% (*p* < 0.001), EC −32.52% (*p* < 0.001), EOL −29.11% (*p* < 0.001), EOR −21.49% (*p* < 0.001); while the CG revealed a small change in EO 1.79%, EC 5.73%, EOL −4.72%, EOR −2.57%.

ANOVA with repeated measures revealed significant differences in the interaction of training type × time between the two groups in: EO [COPL with *p* = 0.019 and COPV with *p* = 0.020], EC [COPL with *p* < 0.05 and COPV with *p* = 0.028], EOL [COPL with *p* = 0.069 and COPV with *p* = 0.069], EOR [COPL with *p* = 0.182 and COPV with *p* = 0.175].

Finally, the time effect in the each conditions showed significant differences in: EO [COPL with *p* < 0.001 and COPV with *p* < 0.001], EC [COPL with *p* < 0.001 and COPV with p < 0.001]; EOL [COPL with *p* < 0.001 and COPV with *p* = 0.002]; EOR [COPL with *p* = 0.012 and COPV with *p* = 0.020].

## Discussion

The aim of this study was to investigate the postural-sway performance of preadolescent male karatekas with specific regard to the effects of a significant increase in their training volume (from 4 to 14 hours a week) as result of a one-week intensive karate training camp. In our study, we used a WBB instead of a force platform, well recognized as a gold standard (Clark et al., 2010). Our decision was taken considering the research hypothesis and the field of intervention. From one side, the ability for a scientist to objectively assess standing balance using a WBB in association with the CoreMeter™ software could provide numerous benefits in a wide range of populations, as showed by our results and the validation study (Clark et al., 2010); on the other side we want to state that the inability of WBB to assess force in the horizontal axes may represent a limitation of this study. Clark et al. (2010) found that the force levels in these two axes only rarely exceeded 5 N. The same authors (Clark et al., 2010) together with Scoppa et al. (2013) demonstrated that although the lack of correction for *x* and *y* axes force is an inherent limitation when deriving COP values from the WBB, its excellent concurrent validity when compared to the gold standard suggests that it is a satisfactory device for assessing standing balance.

Significant differences were observed between bi- and monopodalic open eyes tasks, with the COP length and velocity being significantly greater during this last one for both groups. After cessation of the camp, COPL and COPV changed significantly in the KG when compared to pre-camp values. With regard to the CG, no significant differences in COPL and COPV were observed when compared to pre-camp values. Additionally, values recorded in the CG concerning COPL and COPV were significantly higher than those in the KG.

These results highlight two major findings. First of all, they provide reference values about the postural performance of this athletic population. For example, when compared to the 10 year old non-athletes investigated by Wolff et al. (1998), the karatekas of this study showed a better balance control, as indicated by the lower COPV recorded under the conditions of open eyes (karatekas: 4.43±2.11 vs. non-athletes: 11.3±3 mm·s^−1^) and closed eyes (karatekas: 4.46 ± 1.68 vs. non-athletes: 14.5±3.9 mm·s^−1^) in bi-podalic standing. We therefore suggest that practicing karate can represent a powerful stimulus to the neurological development of balance control in preadolescents. Improvements in postural control in children have been described by decreasing postural sways (Kirshenbaum et al., 2001). As the COP path length and velocity provide indirect information about the balance control process or strategy, the lower values observed in our karatekas indicate an improvement in their postural control when compared to coetaneous non-athletes. In this context, Violan et al. (1997) demonstrated that six months of karate training (two sessions per week) induced a significant improvement in static body balance in 8 to 10 year-old boys when compared to an age-matched group involved in recreational sports. These results as well as those from the current study can lead us to the conclusion that karate training based on exercises, at both long term and short terms, represents a relevant method for increasing human static body balance.

Repeated complex motor tasks, consisting of a variety of bodyweight shifting, body rotation and single leg stances, as performed regularly from the age of 6 to 10 yrs by our karatekas, have probably resulted in building a larger repertoire of postural strategies. These contributed to accelerate the development of balance control. The main reference frame used for the organization of balance control during its development is the pelvis (Assaiante et al., 2005). A regular practice of karate, during a sensitive phase of individuals’ postural-sway development, could therefore improve static and dynamic control of this anatomical structure (Weerdesteyn et al., 2008), thus resulting in an improved balance. In a study comparing body balance between novice (with no experience in martial arts) and expert karatekas (with average years of practice of between 19 and 27 years) while performing two different karate punch techniques, Cesari and Bertucco (2008) reported that expert karate practitioners showed limited backward COP displacement during the punch technique (against a punching box) when compared to novice ones. This result implies better controlling of COP’s migration in expert karate athletes compared to their novice counterparts. Authors concluded that high level karatekas have the ability to perform efficient motor strategies so as to keep their body stable while applying a huge amount of force.

When compared to other athletes, e.g. the 13 year-old soccer players investigated by Bieć and Kuczyński (2010), the karatekas of this study showed a similar postural performance, as indicated by a similar COPV recorded under the conditions of open eyes (karatekas: 4.43±2.11 vs. soccer players: 6.1±2.9 mm·s^−1^) and closed eyes (karatekas: 4.46±1.68 vs. soccer players: 5.7±1.1 mm·s^−1^). Karate and soccer present similar technical components. Indeed, they both require executions of dynamic actions (i.e. kicking) during single leg standing. This could result in similar postural adaptations among young karatekas and soccer players. However, it has to be highlighted that our karatekas were significantly younger than Biec and Kuczyński’s soccer players (2010). As postural control improves during the children’s ontogenesis (Wolff et al., 1998), this indicates that karate could be potentially more effective than soccer in improving postural control at a younger age. However, these hypotheses should be carefully considered due to the differences in the experimental design and the protocol between the mentioned studies. Specific studies, investigating the impact of different sports, on the development of postural strategies in preadolescent athletes are needed to better clarify the activity-related differences in balance control.

The second major finding of this study is represented by the significant improvements in the balance control observed in the KG as result of the intensive training camp. One week of intensive training produced a 37.9% reduction in COPV during open eyes bipodalic standing, as well as in a 29.1% reduction in open eyes monopodalic standing in the KG. To our knowledge, this represents the only study to report a significant positive effect of a short term increase in the volume of regular training on postural control of preadolescent karatekas. This finding is of high interest, both in terms of fundamental knowledge and practical applications. First of all, it could support the hypothesis that preadolescent individuals have a very sensitive neural plasticity. This is described as the brain’s ability to adapt to its environment based on experience and development (Hebb, 2002). This concept translates into the possibility to speed up certain cognitive processes with training or experiences (Galvan, 2010). For example, Rueda et al. (2005) reported that only 5 days of attention training in 4 and 6 year old children resulted in significant improvement of attention. In that case, training had specific effects that were similar to the influence of development.

As adult karate athletes seem to have better balance control (Filingeri et al., 2012; Cesari and Bertucco, 2008), as shown by a reduced cortical reactivity to eyes opening in the condition of a resting state (Del Percio et al., 2007a), it could be hypothesized that a short (1 week) but intense training stimulus (14 vs. 3 hours of regular training), represented by the karate training camp, has resulted in a significant impact on the neurological organization and control of postural performance of our 10 yrs old karatekas. We hypothesized that this improvement was due to the optimization of motor skills which had already been part of our karatekas motor performance. These results are in accordance with those of Fong et al. (2012) who showed that 3 months of taekwondo training can improve sensory organization and standing balance in children with developmental coordination disorders. The main limitations of this study are: 1) the absence of evaluation of dynamic posture; 2) the inability of WBB to assess force in the horizontal axes.

In summary, it may be concluded that short term high intensity karate training can be proposed as an effective method applied in order to improve static balance at an early age. This is relevant in the context of improving the performance of young karatekas in the light of their competitive activity and is also relevant to better understand karate’s mechanisms. As top-level karate performance requires high-levels of both static and dynamic balance, future investigations dealing with the effect of karate practice on body balance should consider the possibility to investigate the dynamic component of postural performances.

## Figures and Tables

**Table 1:** Comparison of data (mean±SD) recorded at the pre and post-test between the two groups

Group	Variable	Pre	Post	(Diff. %)	Group Interaction			Inter. × Time		
		COP Length (mm)			F	p	η^2^	F	p	η^2^
KG	EO	43.42 ± 19.80	27.24 ± 8.38	−37.26^*^	27.878	0.001	0.279	5.789	0.019	0.074
CG		43.73 ± 19.55	45.12 ± 19.90	3.16						
		COP Velocity (mm·s^−1^)								
KG		4.40 ± 2.00	2.73 ± 0.85	−37.92^*^	25.862	0.001	0.264	5.640	0.02	0.072
CG		4.43 ± 2.11	4.51 ± 2.02	1.79						

		COP Length (mm)								
KG	EC	43.39 ± 19.51	29.63 ± 9.45	−31.72^*^	19.412	0.001	0.215	4.100	0.05	0.055
CG		43.97 ± 19.66	44.38 ± 19.82	0.93						
		COP Velocity (mm·s^−1^)								
KG		4.44 ± 1.94	2.99 ± 0.97	−32.52^*^	20.707	0.001	0.223	5.036	0.028	0.065
CG		4.46 ± 1.68	4.71 ± 2.30	5.73						

		COP Length (mm)								
KG	EOL	89.04 ± 33.20	64.76 ± 20.22	−27.27^*^	12.975	0.001	0.158	3.414	0.069	0.047
CG		89.73 ± 34.29	87.01 ± 29.49	−3.03						
		COP Velocity (mm·s^−1^)								
KG		9.19 ± 3.61	6.51 ± 1.99	−29.11^*^	10.554	0.001	0.128	3.250	0.076	0.043
CG		9.17 ± 3.45	8.74 ± 2.96	−4.72						

		COP Length (mm)								
KG	EOR	88.07 ± 30.54	69.19 ± 26.07	−21.44^*^	6.691	0.012	0.086	1.813	0.182	0.025
CG		87.14 ± 31.64	86.30 ± 36.28	−0.97						
		COP Velocity (mm·s^−1^)								
KG		8.80 ± 3.07	6.91 ± 2.60	−21.49^*^	5.672	0.02	0.073	1.876	0.175	0.025
CG		8.78 ± 3.11	8.56 ± 3.59	−2.57						

Values are expressed as mean ± SD for Karate Group (KG) and Control Group (CG). The variables: eyes open (EO), eyes closed (EC), eyes open one leg left (EOL), eyes open one leg right (EOR) was represented between pre and post training with

“*” p < 0.001 versus baseline conditions with group and time interaction

## References

[b1-jhk-38-45] Assaiante C, Mallau S, Viel S, Jover M, Schmitz C (2005). Development of postural control in healthy children: a functional approach. Neural Plast.

[b2-jhk-38-45] Babiloni C, Marzano N, Infarinato F, Iacoboni M, Rizza G, Aschieri P, Cibelli G, Soricelli A, Eusebi F, Del PC (2010). “Neural efficiency” of experts’ brain during judgment of actions: a high-resolution EEG study in elite and amateur karate athletes. Behav Brain Res.

[b3-jhk-38-45] Bauer C, Groger I, Rupprecht R, Gassmann KG (2008). Intrasession reliability of force platform parameters in community-dwelling older adults. Arch Phys Med Rehabil.

[b4-jhk-38-45] Biec E, Kuczynski M (2010). Postural control in 13-year-old soccer players. Eur J Appl Physiol.

[b5-jhk-38-45] Cesari P, Bertucco M (2008). Coupling between punch efficacy and body stability for elite karate. J Sci Med Sport.

[b6-jhk-38-45] Clark RA, Bryant AL, Pua Y, McCrory P, Bennell K, Hunt M (2010). Validity and reliability of the Nintendo Wii Balance Board for assessment of standing balance. Gait Posture.

[b7-jhk-38-45] Cohen J (1988). Statistical power analysis for the behavioral sciences.

[b8-jhk-38-45] Del Percio C, Brancucci A, Bergami F, Marzano N, Fiore A, Di Ciolo E, Aschieri P, Lino A, Vecchio F, Iacoboni M, Gallamini M, Babiloni C, Eusebi F (2007a). Cortical alpha rhythms are correlated with body sway during quiet open-eyes standing in athletes: a high-resolution EEG study. Neuroimage.

[b9-jhk-38-45] Del Percio C, Marzano N, Tilgher S, Fiore A, Di Ciolo E, Aschieri P, Lino A, Toran G, Babiloni C, Eusebi F (2007b). Pre-stimulus alpha rhythms are correlated with post-stimulus sensorimotor performance in athletes and non-athletes: a high-resolution EEG study. Clin Neurophysiol.

[b10-jhk-38-45] Filingeri D, Bianco A, Zangla D, Paoli A, Palma A (2012). Is karate effective in improving postural *control?*. Arch Budo.

[b11-jhk-38-45] Fong SS, Fu SN, Ng GY (2012). Taekwondo training speeds up the development of balance and sensory functions in young adolescents. J Sci Med Sport.

[b12-jhk-38-45] Galvan A (2010). Neural plasticity of development and learning. Hum Brain Mapp.

[b13-jhk-38-45] Hebb BO (2002). The organization of behavior: A neuropsychological theory.

[b14-jhk-38-45] Hrysomallis C (2011). Balance ability and athletic performance. Sports Med.

[b15-jhk-38-45] Hubbard B, Pothier D, Hughes C, Rutka J (2012). A portable, low-cost system for posturography: a platform for longitudinal balance telemetry. J Otolaryngol Head Neck Surg.

[b16-jhk-38-45] Kirshenbaum N, Riach CL, Starkes JL (2001). Non-linear development of postural control and strategy use in young children: a longitudinal study. Exp Brain Res.

[b17-jhk-38-45] Lan C, Lai JS, Chen SY (2002). Tai Chi Chuan: an ancient wisdom on exercise and health promotion. Sports Med.

[b18-jhk-38-45] Leong HT, Fu SN, Ng GY, Tsang WW (2011). Low-level Taekwondo practitioners have better somatosensory organisation in standing balance than sedentary people. Eur J Appl Physiol.

[b19-jhk-38-45] Pau M, Loi A, Pezzotta MC (2012). Does sensorimotor training improve the static balance of young volleyball players?. Sports Biomech.

[b20-jhk-38-45] Perrin P, Deviterne D, Hugel F, Perrot C (2002). Judo, better than dance, develops sensorimotor adaptabilities involved in balance control. Gait Posture.

[b21-jhk-38-45] Rueda MR, Rothbart MK, McCandliss BD, Saccomanno L, Posner MI (2005). Training, maturation, and genetic influences on the development of executive attention. Proc Natl Acad Sci U S A.

[b22-jhk-38-45] Scoppa F, Capra R, Gallamini M, Shiffer R (2013). Clinical stabilometry standardization: basic definitions--acquisition interval--sampling frequency. Gait Posture.

[b23-jhk-38-45] Springer BA, Marin R, Cyhan T, Roberts H, Gill NW (2007). Normative values for the unipedal stance test with eyes open and closed. J Geriatr Phys Ther.

[b24-jhk-38-45] Vando S, Unim B, Cassarino SA, Padulo J, Masala D (2013). Effectiveness of perceptual training proprioceptive feedback in a virtual visual diverse group of healthy subjects: a pilot study. Italian J Public Health.

[b25-jhk-38-45] Violan MA, Small EW, Zetaruk MN, Micheli LJ (1997). The effect of karate training on flexibility, muscle strength, and balance in 8 to 14-year-old boys. Pediatr Exerc Sci.

[b26-jhk-38-45] Weerdesteyn V, Groen BE, van SR, Duysens J (2008). Martial arts fall techniques reduce hip impact forces in naive subjects after a brief period of training. J Electromyogr Kinesiol.

[b27-jhk-38-45] Wolff DR, Rose J, Jones VK, Bloch DA, Oehlert JW, Gamble JG (1998). Postural balance measurements for children and adolescents. J Orthop Res.

[b28-jhk-38-45] World Karate (2013). http://www.thekisontheway.com/leading-sport.

